# Supplementary material for: Identification of photoperiod-induced specific miRNAs in the adrenal glands of Sunite sheep (Ovis aries)
Source: Front Vet Sci. 2022 Jul 22;9:888207. doi: 10.3389/fvets.2022.888207 (PMC9354845; doi:10.3389/fvets.2022.888207)
Supplement: Supplementary file 1 [file Data_Sheet_1.ZIP › Supplementary file/miRDeep2 file.zip!/miRDeep2 file/Novel_Structure/LP42A2/Novel_95.pdf]

Provisional ID	:	NC_019459.2_6800
Score total	:	1.7
Score for star read(s)	:	-1.3
Score for read counts	:	0
Score for mfe	:	1.4
Score for randfold	:	1.6
Score for cons. seed	:	
Total read count	:	57
Mature read count	:	57
Loop read count	:	0
Star read count	:	0

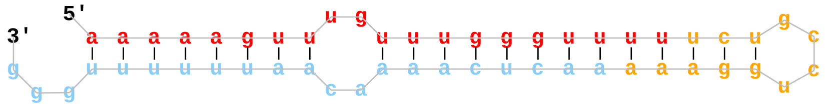

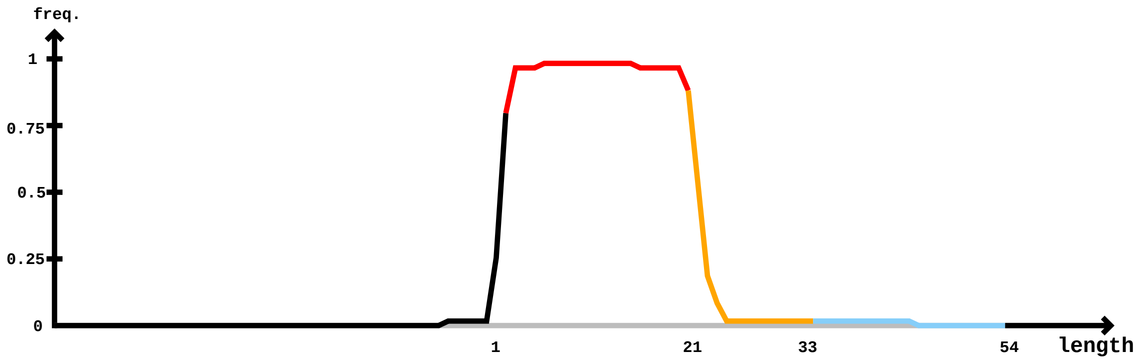

Mature Star

```
5'- aaguguguaguuuuugcauuuaaccucccauauauuggguuggccaaaaaguuuguuuggguuuuucugccuggaaaaacucaaaacaauuuuuugggggccaauucaa  -3'   exp
    (((((((.......))))))).............((((((((((.(((((.(((((((((((((((((....))))))))))).)))))).))))).))))))))))   reads   mm   sample
    .......................................uuggUcaaaaaguuuguuug.................................................   1       1    seq
    ............................................caaaaaguuuguuuggguuuu...........................................   1       0    seq
    ............................................caaaaaguuCguuuggguuuuu..........................................   13      1    seq
    .............................................aaaaaguuCguuuggguuu............................................   5       1    seq
    .............................................aaaaaguuGguuuggguuuu...........................................   1       1    seq
    .............................................aaaaaguuCguuuggguuuu...........................................   24      1    seq
    .............................................aaaaaguuuguuuggguuuuu..........................................   2       0    seq
    ..............................................aaaaguuuguuuggguuuuuc.........................................   2       0    seq
    ..............................................aCaaguuuguuuggguuuuuc.........................................   1       1    seq
    ..............................................aaaGguuuguuuggguuuuuc.........................................   3       1    seq
    ..............................................aaaaguuuguCuggguuuuucu........................................   1       1    seq
    ..............................................aaaaguuuguuuggguuuCucu........................................   3       1    seq
    .................................................aguuuguuuggguuuuucugU......................................   1       1    seq
    ......................................................................cuggaaaaacucaaaaca....................   1       0    seq
```
